# Modulating barriers of tumor microenvironment through nanocarrier systems for improved cancer immunotherapy: a review of current status and future perspective

**DOI:** 10.1080/10717544.2020.1809559

**Published:** 2020-08-31

**Authors:** Huanrong Lan, Wei Zhang, Ketao Jin, Yuyao Liu, Zhen Wang

**Affiliations:** a Department of Breast and Thyroid Surgery, Jinhua Hospital, Zhejiang University School of Medicine, Jinhua, Zhejiang Province, China; b Rehabilitation and Sports Medicine Research Institute of Zhejiang Province, Zhejiang Provincial People’s Hospital, People’s Hospital of Hangzhou Medical College, Hangzhou, China; c Department of Colorectal Surgery, Jinhua Hospital, Zhejiang University School of Medicine, Jinhua, Zhejiang Province, China

**Keywords:** Tumor microenvironment, immunotherapy, nanotechnology

## Abstract

Cancer immunotherapy suppresses and destroys tumors by re-activating and sustaining the tumor-immune process, and thus improving the immune response of the body to the tumor. Immunotherapeutic strategies are showing promising results in pre-clinical and clinical trials, however, tumor microenvironment (TME) is extremely immunosuppressive. Thus, their translation from labs to clinics still faces issues. Recently, nanomaterial-based strategies have been developed to modulate the TME for robust immunotherapeutic responses. The combination of nanotechnology with immunotherapy potentiates the effectiveness of immunotherapy by increasing delivery and retention, and by reducing immunomodulation toxicity. This review aims to highlight the barriers offered by TME for hindering the efficiency of immunotherapy for cancer treatment. Next, we highlight various nano-carriers based strategies for modulating those barriers for achieving better therapeutic efficacy of cancer immunotherapy with higher safety. This review will add to the body of scientific knowledge and will be a good reference material for academia and industries.

## 1. Introduction

Cancer has become one of the world’s most significant health problems. Global population projections have projected rising incidences of cancer over the upcoming years, with 420 million new cancer cases anticipated per year by 2025 (Zaheer et al., 2019). It is traditionally treated with medicines and radiations used to treat anticancer. These therapies, however, are associated with certain disadvantages such as high recurrence possibilities and limited therapeutic efficacy. The clinical intensity of radiation or chemotherapeutic medications at the target sites is accomplished by significant penetration of the majority of the body, contributing to unacceptable side effects (Oshita et al., 1992; Glen and Dubrova, 2012; Huang et al., 2017). Clinicians have treated cancer with assurance in recent years through the use of immunotherapeutic moieties. This strategy also has many benefits including its efficacy against metastasized cancer and low risk of recurrence (Liu and Guo, 2018).

Unlike conventional therapies, immunotherapy targets the immune system to cause systemic therapeutic efficacy. Clinical studies with immune checkpoint inhibitors have demonstrated enormous lasting responses (Farkona et al., 2016; Shi et al., 2018). Specifically, it can inhibit tumor metastasis and relapse by improving the immune system, amplifying the immune response, and triggering immune memory while reducing off-target adverse effects (Shi et al., 2018).

**Figure 1.**
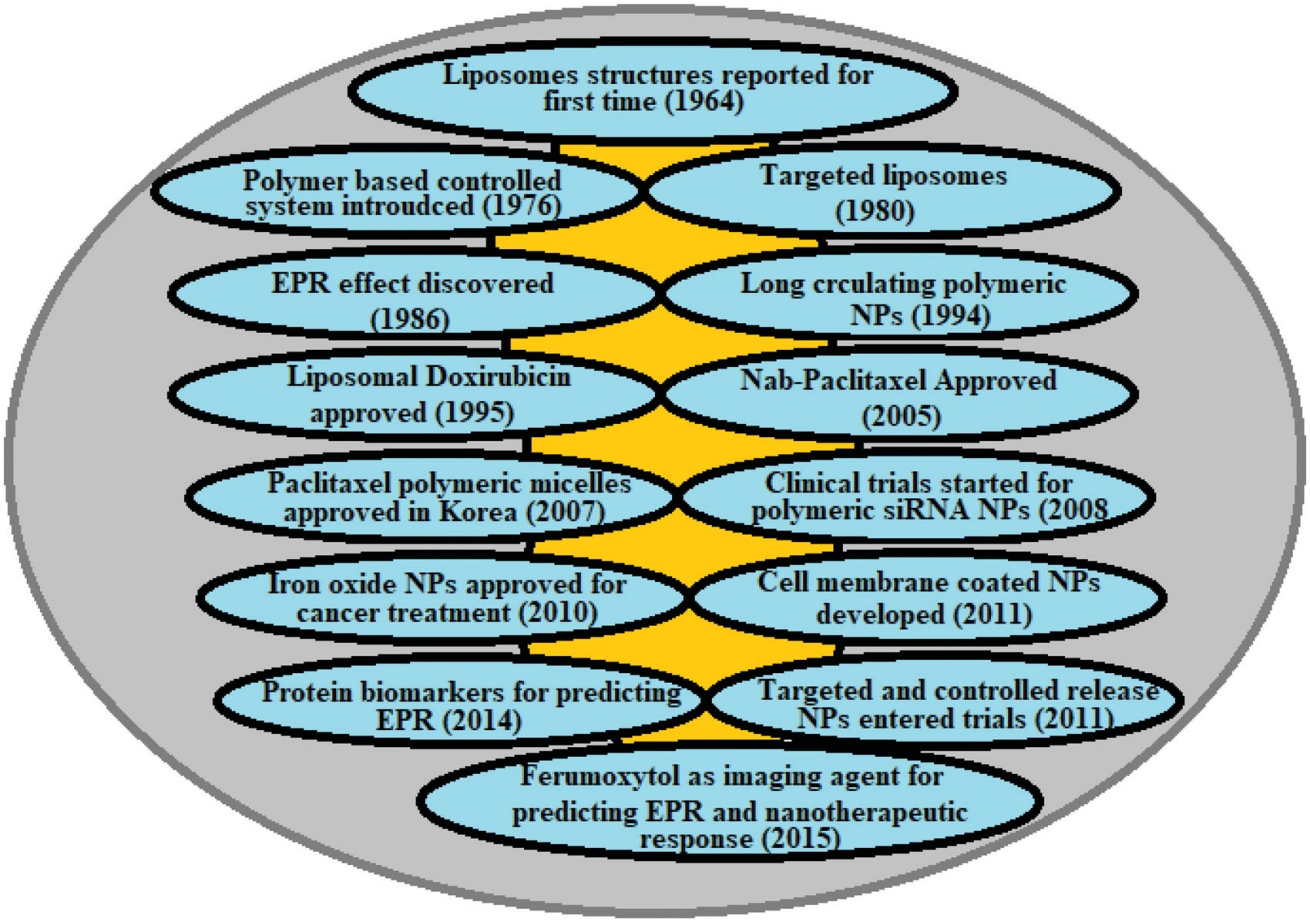
Historical timeline of major developments in the field of cancer nanomedicine.

The Medical Standing Committee of the European Science Foundation states that ‘Nanomedicine is the science and technology of diagnosing, treating, and preventing disease and traumatic injury, of relieving pain, and of preserving and improving human health, using molecular tools, and molecular knowledge of the human body.’ (Webster, 2006) The past years have fueled the formulation and module of a myriad of nanomaterials including, Nanoparticles (NPs) made from noble metals, carbon, heavy metals, etc., in many forms, such as spherical or non-sphere NPs, nanofilms, nanotubes, and nanowires (Chen et al., 2013). Such nanomaterials have special properties that could be investigated for use in theranostics. Carbon nanotubes, for example, are known and reputable, with high durability; iron oxide NPs are superparamagnetic; while gold NPs have distinctive spectral (optical) characteristics (Awasthi et al., 2018). To enhance the therapeutic advantages of nanomedicine, numerous approaches have been developed, particularly active nanomedicine targeting, tumor-responsive nanomedicine, and optimization of nanomedicine’s physiochemical parameters similar to a scale, and charge (Pérez-Herrero and Fernández-Medarde, 2015; Awasthi et al., 2018). With time there are various breakthroughs in the field of nanomedicine for cancer management (Figure 1). Nevertheless, these approaches rely on the advanced production of nanomedicine alone, which cannot resolve the above-mentioned tumor microenvironmental distribution obstacles (Garg et al., 2018). Correspondingly, Tumor Microenvironment (TME) alteration was considered as an effective tool for improving the delivery of cancer nanomedicine (Zhang et al., 2017a).

This review aims to highlight the barriers offered by TME for the efficiency of nanomedicine. Next, we highlight the various strategies to modulate those barriers through NPs and in combination therapy with NPs.

## 2. Challenges offered by TME to nanomedicine

**Figure 2.**
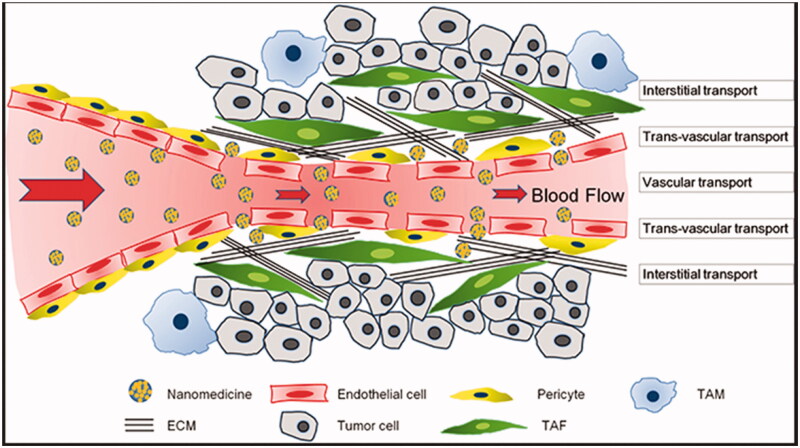
The transport barriers for tumor nanomedicine delivery imposed by a complicated tumor microenvironment (Reproduced from Zhang et al., 2017a).

Given the increasing understanding of tumor growth and advancement, it is practically difficult to ascertain the sequence of actions from the primary phase of tumor production and the unregulated proliferation of cells to a mature high-grade tumor (Feitelson et al., 2015). Occasionally one would be inclined to equate an occurrence induced by the proliferation of tumor cells with an event taking place inside the TME, however, this association is far from the fact because it is the changing interplay of all TME elements that will eventually be responsible for the regulation and development of tumors (Hanahan and Weinberg, 2011; Netea-Maier et al., 2018). TME offers several challenges for the transport of therapeutic material to the site of action and thus hinder the therapeutic efficiency (Figure 2). For simplification, here we will focus individually on each element of the TME. Nevertheless, it is important to note that with a particular tumor or tumor type, any of these events happening with each component may be caused differently, thereby influencing all the other TME components differently, leading to specific outcomes.

The explosive growth of tumor cells leads to such a restriction of the supply of oxygen and nutrients through neighboring blood vessels could not withstand (Hanahan and Weinberg, 2011). The oxygen shortage faced by rising tumor cells induces the cellular reaction to hypoxia, mainly through factors caused by hypoxia (HIF) (Vaupel and Multhoff, 2018). The transcriptional factor HIF family is made up of HIF2, HIF3, and HIF1, proteins that trigger genes involved in the biosynthesis of glucose, angiogenesis, cell proliferation, and migration, and immune response (Graham and Presnell, 2017; Schito et al., 2017). Alongside high energy requirements, the HIF reply in tumor cells induces a metabolic change from oxidative phosphorylation to aerobic glycolysis called the Warburg effect (Gwangwa et al., 2018). Though oxygen presence, this metabolic switch results in increase secretion of lactate into the extravascular environment and corresponding acidification of TME (Lu et al., 2019). The increased proliferation and glycolytic metabolism of tumor cells contribute to a rise in the development of reactive oxygen species (ROS), which in effect attack cellular components, including certain DNA, fostering genomic instability, which affects the morphology of cells, and also stimulates antioxidant capacity (Gwangwa et al., 2018). Such incidents, along with the up-regulation of efflux pumps for the secretion of lactic and carbonic acid, provide a benefit for tumor cells to live and succeed in extreme conditions (Roma-Rodrigues et al., 2019). Intriguingly, the HIF proteins that act as tumor suppressor genes instead of oncogenic promoters in malignant cells (Nakazawa et al., 2016). Nonetheless, HIF-mediated paracrine contact among tumor cells and populations, such as immune system cells, and extracellular matrix modulation and stromal cell metastases, facilitates the growth of tumors and allows HIF proteins oncogenic at TME level (Sormendi and Wielockx, 2018).

The production of vascular endothelial growth factor A (VEGFA) by TME components promotes the growth of adjacent vessels by attaching in endothelial cells to VEGF receptors (VEGFR). The increasing incidence of angiogenic signals at the TME lead in the development of vessels with damaged or undefined basal cells, resulting in the leakage of the vasculature with a disorderly structure unequally applied around the tumor, with cancer areas enriched by vessels and improperly provided cancer areas (Dirkx et al., 2006; Klein, 2018). This restricts the nutrient and oxygen supply to the TME, promoting hypoxia, and difficult the chemotherapeutic agents’ distribution throughout the tumor (Dirkx et al., 2003; 2006). The unstable composition of the blood vessels contributes to the abnormal production of cytokines implicated in inflammatory and coagulation functions at TME (Tei et al., 2002). However, it less structured rusty vasculature enables nanomedicines to selectively attack the source of the tumor. VEGF-D and VEGF-C secreted by cancer cells, stromal cells and immune cells promote the development of lymphatic vessels at TME, termed tumor-associated lymphangiogenesis (Partanen et al., 2000). Thus, lymphatic endothelial cells (LECs) develop single-layer lymph capillaries of the reduced basal lamina, which connect lymph vessels with a basal lamina and valves to avoid regressive discharge (Weitman et al., 2013). Lymphatic vessel development at the TME is associated with a bad prognosis since it promotes metastatic proliferation in distal organs (Albrecht and Christofori, 2011). But on the other hand, LECs play a major role in the regulation of the immune system at TME which contributes to anti-tumor immunity (Weitman et al., 2013; Farnsworth et al., 2014). The faulty lymph drainage once again supports the aggregation of nanomedicines at both the locus by the EPR.

Concerning immune system cells, the TME differs greatly during the production of tumors and across the different kinds of tumors (Netea-Maier et al., 2018). Owing to the constant shifts and modifications that arise at the TME, multiple activation factors (e.g. chemokines and cytokines) are naturally produced, culminating in the identification of cells both from adaptive and innate immune systems (Chen and Mellman, 2013). Notably, TME’s composition of molecular signals influences the therapeutic result by facilitating tumor escape through immunosurveillance or cancer restraint (Gun et al., 2019). Since in the TME, monocytes may distinguish into two major groups of macrophages based on the chemical makeup of the tumor site, M1-type macrophages are produced in the existence of interferon-gamma (IFN-π), and M2-type macrophages just before subjected to various interleukins (IL, e.g. IL-4 or IL-10), translating growth factor-beta (TGF-β), stimulative granulocyte-macrophage colony (Martinez and Gordon, 2014; Mulder et al., 2017). This polarization of macrophages is important for tumor diagnosis and treatment as M1-type is linked with a strong prognosis, whereas tumor-associated macrophages (TAMs) typically has M2 phenotype and lead to metastasis tumor formation, and angiogenesis and invasion (Lim et al., 2017; Schülke, 2018). Inflammation is typically seen in TME, initially stimulated by tumor cells (intrinsic pathway) and maintained and/or exacerbated by other elements of TME (Schülke, 2018). A pro-inflammatory condition normally comes with a bad prognosis (Inácio Pinto et al., 2015). The TAMs-mediated IL-1 cytokine production leads to systemic inflammation and promotes a pro-inflammatory microenvironment (Landskron et al., 2014). The lymphoid descendant cells often have a contrasting function in the growth of tumors. Thus, B cells and regulatory T cells build innate cytotoxic lymphocytes, immunosuppressive microenvironment, and NKT and natural killer cells (NK) cells lead to the immunostimulant TME (Balato et al., 2009; Vivier et al., 2012; Krijgsman et al., 2018). The improved expression of GM-CSF and VEGF induces the formation of myeloid-derived suppressive cells (MDSCs) at the bone marrow, which is deployed to the TME while cells stay undifferentiated (Vetsika et al., 2019; Horikawa et al., 2020). MDSCs are generally associated with bad prognosis as they include angiogenesis and inhibition CD8 + cytotoxic T cells and NK cells (Horikawa et al., 2020).

In epithelial cancers, increasing tumor cells and parts of TME cells are endorsed in an ECM with disorder characterized and biomechanical characteristics comparison with healthy tissues (Brauchle et al., 2018). The reduced oxygenation and inflammatory environment cause changes in ECM proteins that lead to desmoplasia, accompanied by high rigidity (Poltavets et al., 2018) Collagen types I, III and IV, fibronectin, laminin, hyaluronic acid (HA) and osteonectin are the key contributors of ECM to desmoplasia (Mouw et al., 2014).

Stromal cells are often essential to the growth and prognosis of tumors. Because of the inflammatory environment, mesenchymal stromal cells (MSCs) are recruited into the tumor and can facilitate or impede tumor progression as per the chemical composition at the TME (Zhou et al., 2013; Plava et al., 2019). The activation and consequent release of TGF-β in the TME cause the transformation of fibroblasts into fibroblasts consistent with cancer (CAFs) (Sloin et al., 2018). In addition to tumor cells, CAFs are the most prevalent type of cell at the TME and play a significant role in increased TME desmoplasia (Kilari et al., 2018). Enhanced desmoplasia and Hypoxia, interactions between the different TME players facilitate the epithelial-to-mesenchymal transformation (EMT) of tumor cells contributing to the development of stem cells of cancer (SCC) (Martin et al., 2016). EMT leads to disturbance of intracellular adhesion and lack of cell polarity, granting CSC migratory capacity to reach neighboring blood or lymph vessels at TME and move to some other anatomical position when they through undergo mesenchymal-to-epithelial transformation (MET) and potentiate metastatic groove development (Roma-Rodrigues et al., 2019). Matrix metalloproteinases (MMPs) play an essential part in EMT and are responsible for the formation of tumor cells from ECM that promote CSC development (Nisticò et al., 2012; Tsai and Yang, 2013).

In the early stages of tumorigenesis, tumor growth is determined by the genomic makeup of tumor cells. As the tumor grows, TME and tumor advancement are dictated by cell signaling between cancer and surrounding tissue, adding value to intra and intertumor heterogeneous nature (Han et al., 2013; Nielsen and Schmid, 2017). Exosomes are crucial for the interaction between cells. Exosomes are endosomal vesicles with a diameter of 30–100 nm, consisting of a lipid bilayer comprising membrane proteins, trapping soluble proteins, signaling molecules including chemokines, growth factors, and cytokines, and nucleic acids including miRNA and mRNA (Corrado et al., 2013). Notably, the composition of exosomes relies on the precursor cells, which also represents the cell’s physiological response (Simons and Raposo, 2009). Upon release into the extracellular world, secondary cells lateral to the primary cell can embrace exosomes, or migrate across the vascular or lymphatic network to another anatomical place where local cells will internalize them. Ever since internalized, exosomes can modify the participant cell’s phenotype, which could adapt to incoming signals (Mashouri et al., 2019). Tumor cells generated exosomes (TCDEs) play a significant role in the development of tumors, and the regulation of the immune system, leading to the natural tumor movement of neighboring cells and planning the metastatic niche at a newly anatomical position (Mashouri et al., 2019). TME advancement, as mentioned, is remarkably analogous among different cancers, displaying many other similar characteristics in structure and organization. TME characteristics are often highly tissue/organ based, though. Hematological tumors for instance may display the decreased angiogenesis in the bone marrow at the TME area (Han et al., 2016; Zheng et al., 2016).

Given the increasing understanding of the TME’s function in tumorigenesis, tumor development and organism metastasis, report focus to create models, replicate environments, and test experimental drugs. Moreover, many of these showings and evaluations are performed utilizing conventional cell lines which only represent the actual tumor in the persons, leaving the entire heterogeneity of the intra- and inter-tumors benefit of the entire. Among the most pivotal role in the development of biological diagnostics of effects and treatment interventions for tumor has promptly developed suitable results to replace variable and expensive in vivo models (Jean-Quartier et al., 2018).

## 3. Strategies to modulate TME through NPs

**Figure 3.**
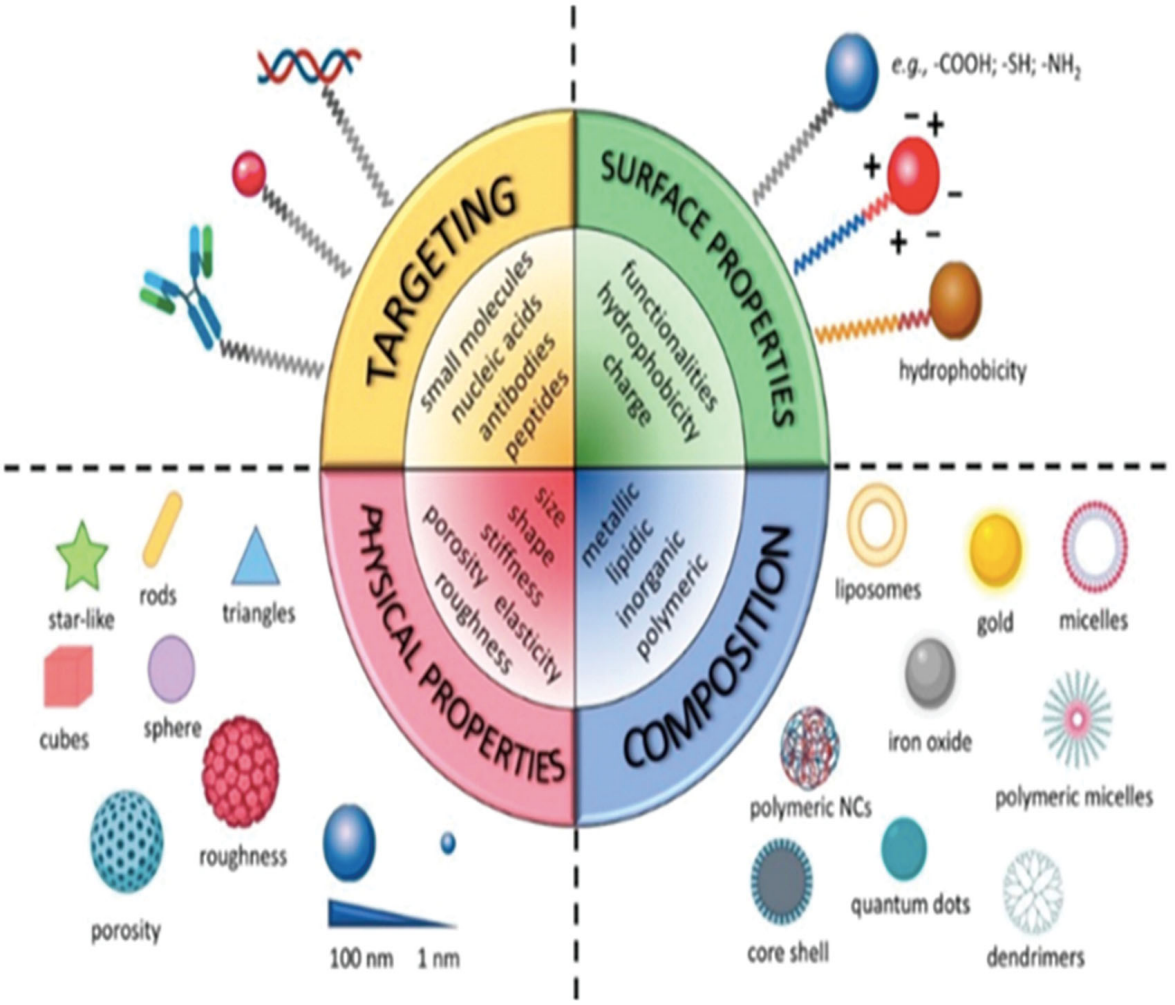
Tunable physical and chemical properties of nanocarriers (NCs) (Reproduced from Salvioni et al., 2019).

NPs targeting systems have proved to be suitable carriers for effective co-delivery of a range of cargoes, including medications, medicinal peptides, and organic compounds. NPs exhibit various tunable properties that can modulate the TME and improve the therapy (Figure 3). The TME’s particular hallmarks, including such weakly acidic pH extracellular matrix, redox potential, hypoxia, etc., were exploited to develop highly targeted delivery systems (Haider et al., 2020). By triggering CTLs, the localized provision of acceptable antagonists and inhibitors can serve as an incentive. Many authors have recently investigated the tremendous potential of engineered NPs to modulate the TME by breaking down potential obstacles (Blanco et al., 2015; Gao, 2016b; Yang and Gao, 2017; Zhou et al., 2020). Some of them are elaborated below.

### 3.1. Reverting immune suppression

Tumors use Immune Control strategies to escape. Many cancer immunotherapies have the principal purpose of maintaining successful immune surveillance. Among many of the numerous processes that control immune escape, tumor microenvironment-associated soluble factors, and/or surface-bound molecules are largely responsible for tumor-specific cell defective behavior (Papaioannou et al., 2016). Such complex immunosuppressive networks inhibit multilevel tumor rejection while reducing immunotherapy performance. Strategies to distribution based on NP allowed immune suppression to be reversed by hindering the IDO pathway. GuangjunNie’s group intended a peptide-assembled nanostructure (DEAP-DPPA-1) usually contains hydrophobic and hydrophilic domains where PLGLAG, a peptide substratum of abundantly expressed proteinases, e.g. matrix metalloproteinase-2 (MMP-2) and functional 3-dimethyl aminopropyl isothiocyanate (DEAP) were combined to make a hydrophobic domain, whereas the hydrophilic domain consisted of a peptide antatic domain (Tang et al., 2013). Within physiological conditions the administered NLG919, a well-known IDO inhibitor, co-assembled into micelle-like NPs. The undistributed hydrophobic nucleus of NPs in the acidic tumor niche allowed MMP-2 to hydrolyze and cleave the peptide substratum and start releasing modified DPPA-1 and NLG919 accurately (Saeed et al., 2019). The controlled release of updated DPPA-1 and NLG919 meant that immunosuppressive channels, such as PD-L1 and IDO, were inhibited, simultaneously and eventually rescued. After treatment, the levels of IFN-ÿ and IL-2, and the amount of NK cells were increased (Guerrouahen et al., 2019).

### 3.2. Improving permeability through inflammatory mediators

Inflammatory mediators including TNFα, prostaglandin analogs, VEGF, and nitric oxide (NO) donors, which are capable of improving vascular permeability, were reported to improve the concentration of nanomedicine in tumors up to 2-6 times more than the control group (Abdulkhaleq et al., 2018). In addition to increased vascular permeability, vasodilatation, and optimizing blood flow by the use of inflammatory mediators have led to improving the production of nanomedicine for cancers (Nehoff et al., 2014). Nevertheless, a sequence of actions of the above listed inflammatory mediators may also contribute to increased IFP against the delivery of nanomedicine. The accretion of nanomedicine in cancers is therefore highly based on such variables. Because inflammation may potentially facilitate the growth of cancer cells, local application (or direct distribution to the tumor cells of inflammatory mediators) must be incorporated (Dawulieti et al., 2020).

### 3.3. Vessel normalization strategy to improve the delivery of nanomedicine

Various approaches are adopted to manipulate the blood and tumor vessels to modulate the TME and facilitate drug delivery. Some of them are discussed below.

#### 3.3.1. Normalization of tumor vasculature

**Figure 4.**
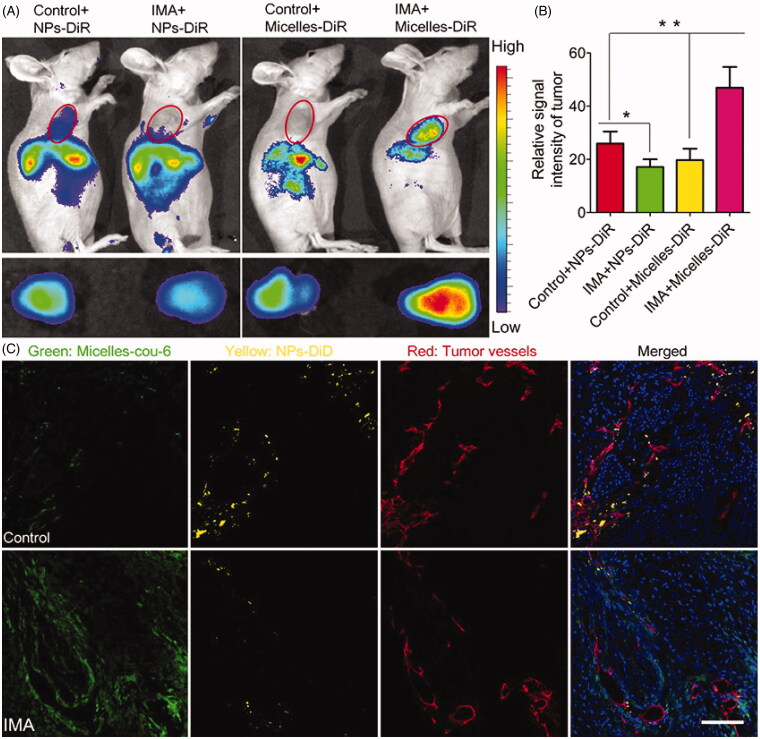
The effects of IMA treatment on tumor NP delivery. (A) *In vivo fluorescence imagery of A549 xenograft-bearing mice (upper row) treated with IMA or water as a buffer, ex vivo fluorescence imagery of their respective xenografts (lower row)*, and (B) Relative tumor tissue signal intensity 24 hours after DiR-labeled NPs or micelles are injected. *p < .05, compared with Control + NP group. **p < .01 compared with the IMA + Micelles group. (C) In vivo dissemination of micelles and NPs from A549 tumor xenograft-bearing mouse models treated with IMA or water 24 h after i.v. in tumor slices Injection of a combination of DiD-labeled NPs and micelles labeled with coumarin-6. During 3 weeks the oral dose of IMA was 50 mg/kg/d. All the coumarin-6 and DiD concentrations were 0.05 mg/kg. 100 μm was indicated in the slot (Reproduced from Zhang et al., 2016).

The freshly developed tumor vessels are still tortuous and leaky, enabling both for extravasation of nanomedicine while at the same time the IFP, which inhibits sufficient and homogeneous nanomedicine blood circulation and systemic flow (Chen et al., 2017). Standardization of the vessels has emerged as an important solution to optimizing nanomedicine distribution for tumor therapy. Regularization of the vessels turns the pathological phenotype of the tumor vessels into a phenotype that strongly matches that of normal completely functioning vessels by restoring the basal layer and growing pericyte distribution and eventually reducing vessel leakage (Zhang et al., 2019; Wang et al., 2020). Increasing the tumor vessel architecture could greatly reduce fluid extravasation and lower IFP, and instead restore tumor blood flow, thus working to improve nanomedicine vascular transport. Several proangiogenic molecules, such as VEGF, fibroblast growth factor (FGF), and PDGF, are over-expressed in tumors and engaged in angiogenesis, which induces disorderly structural formation in such freshly developed tumor vessels (Zhao and Adjei, 2015; Zhang et al., 2016). Consequently, techniques were developed to suppress these proangiogenic signaling molecules and restore tumor vessels. In the diagnosis of metastatic colorectal cancer, for example, VEGF inhibitors Bevacizumab, the FDA-approved antiangiogenic monoclonal antibody (mAb), capable of restoring irregular tumor vessel configuration to a more natural phenotype, is added (Kong et al., 2017). It has culminated in the creation of therapies to inhibit these proangiogenic signaling molecules and rebuild tumor vessels. In the treatment of metastatic colorectal cancer, for example, VEGF inhibitors Bevacizumab, an antiangiogenic monoclonal antibody (mAb) approved by the FDA, which can return abnormal tumor structure to a more normal phenotype (Saxton and Sabatini, 2017), Notch 1 signaling (Lee et al., 2015), and D2 receptors-angiopoietin 1 signaling (Chauvet et al., 2017). Often interested in vessel standardization, to boost the production of nanomedicine. By previous work, imatinib mesylate (IMA) has also been shown to normalize tumor vessels of A549 tumors by preventing the signaling mechanism of the platelet-derived growth factor (Zhang et al., 2016). Interestingly, treatment with IMA could significantly reduce the aggregation of NPs (NPs) by about 110 nm but increased the accumulation of micelles by about 23 nm. In comparison, IMA therapy reduced the distribution of NPs within tumors but enhanced that of micelles with a more homogeneous pattern (Figure 4) (Gao, 2016a; Zhang et al., 2016). Eventually, the anti-cancer effectiveness analysis found that pretreatment with IMA could substantially increase the therapeutic impact of paclitaxel-laden micelles. As tumor vessel normalization narrowed endothelial space, tumor cells could be stopped from splitting into tumor vessels, and tumor metastasis could be decreased to some degree.

Four concerns must be taken into consideration to use vessel normalization strategy to improve the delivery of nanomedicine for tumor treatment. First, the technique will only boost the distribution of low molecular weight drugs or comparatively smaller nanomedicines varying from 20 to 40 nm but reduces the transmission of large nanomedicines by about 100 nm because it eliminates cancer vessel endothelial gaps (Mattheolabakis and Mikelis, 2019). Second, the regularization is reversible and the nanomedicine that occurs will be introduced in the normalization cycle (Chauhan et al., 2012). Third, it is strongly advised that a judicious dosage of vascular normalizer avoids unnecessary pruning of tumor arteries, which may impede vascular capacity and therefore the transmission of simultaneous therapy (Cheng and Saltzman, 2012). Fourthly, provided that vasculatures are still extremely cramped in strongly desmoplastic tumors and refractory to vasculature normalizers, this technique should either be used during tumors that are relatively porous and not extremely desmoplastic or at least paired with the other techniques that can restart crushed vessels (Jiang et al., 2015).

#### 3.3.2. Tumor vessel dilation

Vasoconstrictive endothelin-1 (ET1) and its ETA receptor, by which ET-1 mediates vasoconstriction, are also present in cancer cells to preserve the tumor vessel contractile signal. The frequency of expression of ET1 and ETA in tumor vessels was 13 and 5 times greater than that of regular vessels balanced in scale, accordingly (Kowalczyk et al., 2015). BQ123, a selective antagonist against ETA, can prevent ET1-ETA signaling, stimulate dilation of the tumor vessels, and trigger tumor-specific blood flow growth. The BQ123-induced boost in the blood flow increased the distribution of free drugs to tumors following a rise in IFP (Zamora et al., 1993). Furthermore, it has been shown that BQ123 could promote the levels of photothermal nanomedicine by about 100 nm for successful photothermal treatment of tumors (Zamora et al., 1993). A few inflammation variables, including such bradykinin, which can dilate vessels, also could boost tumor perfusion directly. In our previous research, captopril, a commonly used hypotensor in clinics, has been shown to dilate tumor blood vessels by growing the bradykinin expression and also growing the permeability of tumor vessels to increase the distribution of nanomedicine for cancer treatment (Zhang et al., 2017a).

An IDO pathway is designated as a crucial immunosuppression regulator. Engineered NPs allowed immune suppression to be reversed by blocking the IDO pathways (Mbongue et al., 2015). The findings indicate that the NP dependent immunostimulatory formulations have a strong antitumor effect (Feng et al., 2019). Feng et al. (2018) demonstrated that The TME can be mediated by constructing a dual-activatable (acidic and reduced) binary cooperative drug NP(BCPN) in which amphiphilic oxaliplatin (OXA) and NLG919 have been assembled for enhanced immunotherapy treatment. BCPN assured increased absorption and penetration attributable to charging reversing characteristics of deshielded polyethylene glycol (PEG) in acidic TME, whereas OXA prodrug and NLG919 were enabled in the TME to minimize. BCPN can cause ICD successfully and can reverse the immunosuppressive process due to the involvement of OXA and NLG919, collectively. The apparent rise in apoptosis of tumor cells and reduce in tumorigenesis were ascribed to the activation of DCs, CTLs, and pro-inflammatory cytokines (IFN-ÿ). NLG919 has also been found to be beneficial in suppressing intratumoral Treg cell infiltration

### 3.4. Improving transvascular delivery of therapeutics to TME

For strongly desmoplastic tumors, the pericyte penetration levels on endothelium were around 70%, far higher than porous and permeable tumors, greatly restricting nanomedicine’s transvascular movement through tumor interstitium (Aguilera and Brekken, 2014). Strategies were then formulated utilizing a low dose of a TGF-β receptor, LY364947 to decrease the pericyte distribution of endothelium and to increase the size differences between endothelium to improve the therapeutic benefits of gemcitabine-charged liposomes for pancreatic cancer and Doxil for dispersing gastric tumor (Miao et al., 2015).

Platelets are widely believed to lead a great deal to hemostasis. Besides its position in the creation of thrombosis, platelets are also deeply engaged in tumor progression and metastasis. Additionally, it may also help tumor vascular homeostasis and preserve tumor vessel integrity (Gay and Felding-Habermann, 2011). The research found that the removal of platelets caused bleeding at the tumor site and decreased tumor vasculature leakiness. Platelet elimination in thrombocytopenic mice also improved the effectiveness of breast cancer therapies (Wang et al., 2018b). To avoid potential bleeding in normal organs caused by low platelet counts, a recent study by Li et al. (2017) designed a tumor microenvironment-responsive NP worthy of distributing antiplatelet antibody R300 to specifically reduce platelets in cancer cells, thereby increasing endothelial dysfunction and enhancing the distribution of nanomedicine to tumors. Platelet reduction was a viable candidate for increased transvascular nanomedicine delivery to tumors.

### 3.5. Targeting lymph nodes targeting strategy to modulate TME

Although all of the NP structures either spread in the bloodstream or are meant to concentrate in the tumor, attacking the lymph nodes is another critical field where NPs may have a major effect. Irvine et al. have reported many papers on NPs in the polymer that travel to lymph nodes (Liu et al., 2014). To provide higher therapeutic benefits on cancer vaccines, vaccine adjuvants should accrue in lymph nodes, in which naïve T and B cells are fully prepared. CpG is a DNA sequence that binds TLR9 and may be a strong immunostimulant, but free CpG does not concentrate on lymph nodes. Irvine et al. conjugated CpG to a lipophilic albumin-binding domain and demonstrated that such peptide vaccines travel to lymph nodes through albumin hitchhiking, based on the nanoparticle. One week after injection, the concentration of albumin-binding CpG-liposomes was 6 times higher than those of soluble CpG in lymph nodes but this mechanism also contributed to a persistent regression of tumors in murine melanoma models (Liu et al., 2014; Mehta et al., 2015). Injecting vaccines into lymph nodes improves the efficacy, however timely clearance of vaccines remains a concern. To overcome this problem, Irvine et al. merged nanoparticle-based vaccinations with intralymph node injections. The combination of intralymph node vaccination strategies with a PLGA micro- or nanoparticular-conjugated TLR3 agonist improved lymph node aggregation, enhanced T-cell cytokine development and resulted in more sustained DC stimulation in immunized mice (Andorko et al., 2014; Mehta et al., 2015).

Through comparison, in tumor-bearing mice, Swartz et al. inserted lymph node-targeting nanoparticle-conjugated TAA and adjuvant intradermally. Those NPs collected efficiently in the lymph nodes given the particular distribution pathway. Besides that, once bonded to NPs and inserted into the TAA-primed tumor-draining lymph node the vaccine had stronger therapeutic effects. Following vaccine administration, the immunosuppressive condition of the tumor-draining lymph nodes was restored toward a more immunogenic setting (Maisel et al., 2017). Swarz et al. have used pyridyl disulfide NPs aimed at tumor-draining lymph nodes to distribute hydrophobic DC inducing agents like TLR9 agonist and TLR4 agonist paclitaxel. They demonstrated higher DC ripening, additional production of IL-12, and slower tumor growth utilizing this delivery method (Stewart and Keselowsky, 2017). Similar findings suggest that functionalized NPs are capable of transmitting adjuvant cancer vaccines to lymph nodes and growing immune responses, using a range of distribution routes. Using NPs can boost the adjuvant’s circulation time due to the complex’s larger size, and functionalized particles could even specifically attack crucial areas like the lymph nodes.

### 3.6. Physical stimulus

Radiation may enhance the distribution of nanomedicine intended for tumors (Xin et al., 2017). Several potential pathways are as follows: firstly, through triggering the hypoxia-inducible factor 1 (HIF1), radiation may regulate cell the amount of the vascular endothelial growth factor (VEGF) (Moeller et al., 2004) or via numerous mitogen-activated protein kinase reliant paths to boost tumor vessel permeability. Evidence indicated that perhaps the permeation of magnetic resonance contrast agent with molecular weight over 200 kDa by the cancerous cells was improved by 32.8% after irradiation (10 Gy) (Reitan et al., 2010). Furthermore, radiation will easily destroy the cells of the susceptible tumor. The decreased cell density helped to alleviate tension burden from tumor cells, reopen closed arteries and thereby improve the blood supply of tumors (Delarue et al., 2014). The impact of radiation on tumors is dynamic and depends on the amount of dosage, duration, and tumor (Dolega et al., 2017). Milosevic’s recent review provides further evidence of the same

Koning et al. (2010) pioneered Enhanced vascular permeability with the usage of moderate hyperthermia (HT) for nanomedicine extravasation of tumor tissues. Research has also shown that mild HT may also help increase tumor perfusion and decrease IFP, potentially by vascular fenestration and vascular endothelial disruption, thus allowing for deep nanomedicine infiltration into the cancers instead of perivascular aggregation (Stylianopoulos, 2017). There has been, nevertheless, no clear evidence to prove the tumor vessel pore size change after HT treatment. The extent and severity of extravasation in tumor interstitium were known to differ widely between cancer cell forms, which depends on the morphology of the endothelial lining and the intrinsic properties of the underlying tumor microenvironment, such as interstitial matrix composition. Highly desmoplastic tumors, other than cancers of a certain vascular component, also could react well with mild HT therapy (Golombek et al., 2018). Temperature is a key component in the heat source, in which data showed that 41–43 °C was suitable that a very maximum temperature could harm the tumor vessel’s endothelial lining and stimulate response to coagulation. The creation of thrombins could choke the vessels and negotiation the delivery of nanomedicine. Conversely, inadequate temperature could have a limited impact on the endothelium of the tumor vessel to raise the endothelial gap (Cicha, 2015; Karagkiozaki et al., 2016).

Ultrasound was used to boost the transmission of nanomedicine to tumors through both mechanical and HT impact (Tharkar et al., 2019). For structural results, several studies have shown that gas-filled bubbles may be used to transiently create pores in blood vessels or cell membranes (sonoporation) through which nanomedicines of various types may easily extravasate tumor vessels or penetrate tumor cells, thereby enhancing nanomedicine distribution (Han et al., 2017). Besides, ultrasound also generates energy at a time-dependent, acoustic intensity. Frazier previously using magnetic resonance imaging-guided, high-intensity oriented ultrasound (HIFU) to achieve a nearly uniform heating pattern of 43 °C in a xenograft tumor model and increased the aggregation of Evans blue dye in warmed tumors to nearly 2-fold better than those in unheated cancers (Hijnen et al., 2014)

### 3.7. Companion diagnostic

The companion diagnosis, which corresponds to a patient stratification dependent on tumor properties, is a compelling approach to boost the effectiveness of nanomedicine. There are presently numerous approaches under review, focused on the usage of biomarker signatures and image evidence (Hare et al., 2017). The very first sought to assess TME-associated circulatory proteins highly linked to the EPR effect. For example, the proportion of MMP9 to the metalloproteinase tissue inhibitor 1, the collagen content in the capillary walls, and other angiogenesis markers has also been shown to forecast the EPR agent (Salvioni et al., 2019). But on the other side, radio-labeled and ferumoxytol-charged NCs are being adopted to supervise their bioavailability using noninvasive techniques (e.g. Tomography and magnetic resonance imaging measured single-particle emission or electron absorption, respectively). The finished step is to obtain students with the best probability of responding positively to a particular clinical intervention (Greish et al., 2018). These methods, though, should be further tested by reliable correlative tests, establishing a consistent range of parameters and requirements that can forecast the clinical result. Table 1 summarizes TME modulation strategies for improving tumor nanomedicine delivery.

**Table 1.** Summary of TME modulation strategies for improving tumor nanomedicine delivery.

Modulation approach	Working mechanism	Agents	Tumor model	Reference
Improving interstitial transport	Reprogramming or depletion of TAF	Quercetin NP downregulating the expression of Wnt16	Bladder tumor	Hu et al. (2017)
		Losartan	Human pancreatic, skin and breast tumors	Diop-Frimpong et al. (2011); Chauhan et al. (2013)
		VDR ligand	Pancreatic cancer	Sherman et al. (2014)
		ATAR	Pancreatic cancer	Chronopoulos et al. (2016)
	ECM degradation	PEGPH20 (PEGylated hyaluronidase)	Pancreatic cancer	Hingorani et al. (2016)
		Matrix metalloproteinases-1 and − 8	Sarcoma	Mok et al. (2007)
		rtPA	Melanoma and Lung cancer	Kirtane et al. (2017)
	ECM reduction through inhibiting TAF activity	Cyclopamine	Pancreatic cancer	Jiang et al. (2017)
		IPI-926	Pancreatic cancer	Olive et al. (2009)
Advancing tumor perfusion	Tumor vessel dilation	BQ123	Colorectal carcinoma	Wang et al. (2017)
		Captopril	Glioma	Zhang et al. (2017b)
	Tumor vessel normalization	Chloroquine (Notch 1 signaling inhibition)	Melanoma	Maes et al. (2014)
		Imatinib mesylate	Lung cancer	Zhang et al. (2016)
		DC101	Colon adenocarcinoma, small cell lung carcinoma, glioblastoma multiforme, Mammary carcinoma,	Tong et al. (2004)
		Rapamycin	Melanoma	Guo et al. (2014)
		Dopamine	Colon and prostate tumor	Chakroborty et al. (2011)
Improving nanomedicine extravasation	Platelet depletion	R300 (Antiplatelet antibody)	Breast cancer	Li et al. (2017)
	Inflammatory mediators for enhancing vessel permeability	VEGF	Colon and Glioma carcinoma	Monsky et al. (1999)
		TNF- alpha	Melanoma and lymphoma	Curnis et al. (2002)
		Prostaglandin	Hepatocellular carcinoma	Tanaka et al. (2003)
	Pericyte depletion by inhibiting TGF signal pathway	TGF- type I receptor (TR-I) inhibitor	Gastric cancer, Pancreatic cancer.	Kano et al. (2007)
		A small-molecule TGF-β inhibitor, LY364947	Pancreatic cancer	Meng et al. (2013)
		ID11 (anti-TGF-β mAb)	Breast cancer	Liu et al. (2012)

## 4. Nps in combination therapies for modulating the tumor microenvironment

After a mixture of traditional treatments, such as surgery, radiotherapy, and chemotherapy, cancer relapses contribute to clinical failure. The implementation of new approaches to fight cancer is crucial. Specific approaches will be based on increasing immune reaction, which will stimulate immune memory to resolve the cancer relapse (Subhash et al., 2015). NPs can be used in a joint venture with different therapeutic approaches to modulate the TME. Some of them are discussed below.

### 4.1. Nps with immunotherapies to modulate TME

ICD-inducing chemotherapeutic entity, such as doxorubicin, oxaliplatin, and cisplatin, was delivered with NP-based formulations, resulting in complementary immunotherapy reactions when coupled with IDO inhibitor (indoximod) or immune control blockade (Wang et al., 2016). Differential diagnoses, like chemo-PDT therapy, are not successful toward metastasis; nevertheless, the mixture of chemo-PDT therapy with checkpoint blockade therapy could not only prevent tumor growth and also display promising results toward cancer growth owing to the reverse of T – cells exhaustion (Agostinis et al., 2011). Chlorine e6 and doxorubicin-loaded hollow manganese dioxide nano platform (H-MnO2-PEG/C&D) will alleviate tumor suppression, although checkpoint blocking (PD-L1 blocking) promotes higher TNF-α secretion and improved immune response of CD4 + and CD8 + T cells that chemo-PDT therapy (Yang et al., 2017). For enhancing the ability of immunotherapy, Zhou et al. designed the nanoplatform by combining the OXA prodrug and PEGylated Photosensitizer (PS) that induced ICD and cancer cell phagocytosis by blocking CD47. The application of ICD activation and CD47 blockade strengthened T-cell – mediated reaction, DC maturation tolerance to antitumor, resulting in clinical outcomes, tumor metastasis, and tumor relapse prevention (Gao et al., 2019). Lymphatic metastasis inhibition was due to combined therapy in the B16‐F10 model (Potez et al., 2018). An apparent rise in recruitment of DCs, tumor-infiltrating CTL (CD8+ and CD4+), and as well as a substantial decrease in Treg cells is reported when paired with PDT immunotherapy. As a result, more than 90% of tumor growth was due to iron NP-based relaxed immunosuppression and penetration of T cells (Chen et al., 2016).

TLR agonists and control-point blockade strategies are combined with photothermal and radiotherapy to achieve effective therapeutic efficacy and immunological memory (Chen et al., 2016). The integration of the NP-based sonodynamic treatment framework of immunotherapy blockade and immune adjuvant avoids tumor metastasis and induces an antitumor and immune response by inducing robust immune responses, including increased maturation of DCs, CD4 + and CD8 + lymphocyte infiltration, CD45 + leucocytes, and cytokine secretion (Saeed et al., 2019). The integration of NP-based immunotherapy with other treatment regimens will also unlock the capacity for cancer therapies. Even so, a fuller knowledge of the factors involved in the immune systems and the NPs-mediated toxic effects must be given immense attention (Saeed et al., 2019). Furthermore, NPs are also reported to modulate the TME and improve the efficiency of CAR-T therapy. It can ease the manufacturing process of CAR-T cell production and modulate to complex solid TME to enhance the efficiency of therapy (Nawaz et al., 2020).

Perhaps the more productive method to anticancer treatment is to target a combination of the TME’s vascular, ECM, and immune cells and the actual tumor cells as well. Liu et al. conducted a study wherein liposomes have been used to encompass anti-VEGF agents and adorned via an antagonist CXCR4 to attack all angiogenic and immune responses in a model of hepatocellular carcinoma (Martin et al., 2020). CXCR4 is abundantly expressed both in cancer and immune cells inside the TME and acted as both the targeting ligand and the immune response modulation process (Xu et al., 2015). These CXCR4-targeting liposomes were first compared with sorafenib, a currently licensed anti-VEGF small-molecule medication, and combined therapy was shown to be more successful than both therapies alone. Those who then substituted sorafenib to anti-VEGF siRNA, load current combination with the targeting liposomes delegated authority vessel density and inhibited tumor growth, and prevented TAM from infiltration into the cancer cell (McCallion et al., 2019). NP-based approaches may be implemented to leverage the ICD-inducing properties of traditional therapies to enhance cancer immunotherapy’s therapeutic ability. Therapeutic agents focused on NP, including phototherapy, photodynamic therapy, radiation therapy, and immunotherapy chemotherapy enhance clinical efficiency by allowing the concurrent distribution of different therapeutic substances (Lim et al., 2019; Wu et al., 2020).

### 4.2. Nps in combination with the drug for TME modulation

In addition, drug combination can attack both the TME and the tumors cells oneself. A further cohort reported recently a multivolume nanocarrier capable of delivering multiple anticancer agents and assembling them within the TME into ‘drug delivery depots.’ Whose pH-sensitive carrier produced HA, that traffic toward tumor cells overexpressing both the CD44 HA receptor and hyaluronidase (HAase). As near to the tumor site, HAase cleaves HA, which causes the crosslinking of certain nanocarrier elements, creating depots that are slowly destroyed by the TME acidity. Once packed with TNF-related apoptosis-inducing ligand and anti-angiogenic drug cilengitide, such carriers resided at the tumor site, collated in depots, continued cargo activation, decreased tumor vascularization, and slowed significantly tumor growth without adverse side effects. Furthermore, those who recommended that this platform for NPs can also be used to carry a wide range of cargo like small-molecule chemotherapeutic agents and immune modulators (Hu et al., 2016). Finally, Jiao and coworkers used drug combination in tumor theranostics and often has medicinal advantages for a diagnostic agent. They paired gold NPs with a chimeric tumor binding antibody, anti-GD2, adjusted to improve NK cell activity by interacting with the Fc receptor. The NPs successfully trafficked to cancer cells expressing GD2 and increased computed tomographic contrast, so even small tumors had been visible on diagnostic scans. Antibodies Fc regions bound to the Fc receptors on NK cell surfaces and induced an immune response to cancer cells. Intriguingly, once conjugated with the NPs, the antibodies had a larger impact on NK stimulation than it was when using individually, likely owing to the arrangement of several antibodies linked from each NP (Jiao et al., 2016). Thereby, drug combination can significantly impact numerous facets of TME, target tumor cells and TME, or provide both diagnostic and therapeutic impacts.

### 4.3. Np based combined chemotherapies to modulate TME

Damage-associated molecular patterns (DAMPs) are revealed to the surface by dying/stressed cells, released or secreted. DAMPs, including surface-exposed CRT, passively released HMGB1, secreted ATP, and heat-shock proteins that may act as either hazardous signals or immune system adjuvants to induce ICD in cancer cells (Krysko et al., 2013; Land, 2015). When tumor cells die, DAMPs are emitted that can serve as a warning or combinatorial code to activate various inflammatory cells. ICD adjuvants, like chemotherapeutic agents (anthracyclines and oxaliplatin) and photodynamic therapy (PDT), may activate immunologic apoptosis where dendritic cells swallow their bodies and present T-cell tumor-specific antigens to cause an immune reaction to antitumor (Hou et al., 2013).

By inciting the threat signaling pathways, ICD may be caused by the generation of ROS and endoplasmic reticulum tension (ER). The pressure-specific pathways include the release of main DAMPs, including CRT and ATP (Farooqi et al., 2015; Cubillos-Ruiz et al., 2017). Thus, PDT may cause ICD in cancer cells by inducing ER stress-dependent on ROS. Cells treated with anthracycline will also cause ICD and provoke an immune reaction to the antitumor without any adjuvant. The tumor cells which experience ICD may cause CRT to be translocated on their cell membrane, which eventually results in tumor-specific cytotoxic T lymphocyte-mediated immune responses (Zhou et al., 2019). While PDT and chemotherapy that activate an anti-tumor immune response, T cell fatigue significantly impairs the mediated immune response by PD-1/PD-L1 up-regulation. T cell exhaustion may be altered by obstructing the pathways to the immune control point (for example PD-1) (Asadzadeh et al., 2020). Immunotherapy with a blockade at the immune control point could be used to measure the intensity and usability of tumor-specific T cells to intensify antitumor effectiveness (Wang et al., 2018c).

Wang et al. (2013) designed combining several features including ultra-pH-sensitive diblock (PDPA), and siRNA and pheophorbide A photosensitizer (PPa), may improve therapeutic ability. Hydrolyzed amphiphilic polycation, e.g. 1,2-epoxytetradecane alkylated oligoethylenimine (OEI-C14), having an intrinsic binding affinity with siRNA, thereby promoting proton sponge to ensure the endosomal escape of siRNA. PDPA-OEI-C14-PPa (POP) micelleplex was activated precisely in acidic pH (6.2) while maintaining an intact epithelial microenvironment. The pdna-PD-L1-conjugated micelleplexes (POP-PD-L1) efficiently triggered blockade of PD-L1 and therefore rescued the tumor cells from immunosuppression through silencing the expression of PD-L1. The immune reaction to antitumor was intensified by photodynamic therapy (PDT), where it effectively eradicated the tumor and remote metastasis in the B16-F10 melanoma model through encouraging cytokine production (TNF-α and IFN-ÿ) and tumor invasion lymphocyte frequency (CD8+ and CD4+). Within a week of combination therapy, its most apoptotic cancer death was observed than individual modalities and was thus ultimately caused by the existence of activated immune cells.

Doxorubicin-charged lipoprotein-mimicking nanodiscs (sHDL-DOX) may impose antitumor effectiveness by activating tumor cells ICD (Kuai et al., 2018). Composite chemoimmunotherapy may instruct cancer cells to blockage of the immune checkpoint and potentiate the cell-mediated response to antitumor T. Through delivering chemotherapeutic agents via nanodisks, the removal of therapeutic action (MC38 and CT26 colon carcinoma) in 80–88% of animals was achieved. Survivors are shielded from tumor relapse because of the mediated antitumor memory (Wang et al., 2018a).

### Conclusion

TME has also been involved in growing and metastasizing cancer. With-tumor awareness, tumors are shown to develop in increasingly heterogeneous but diverse microenvironments composed of ECM elements, immune cells, vasculature, TAMs, and CAFs. Recent advances indicate that TME modification as well as its unusual structure is an effective technique for curbing tumor growth, invasion, and metastasis. New strategies for addressing the increasing cancer challenge have grown with the introduction of nanotechnology in the drug discovery field. Even so, the sophistication of the TME has already shown an important so far provocative role in the regulation of deeper nano-chemotherapeutic tumor absorption and consequently its biochemical mechanisms. Strategies have been proposed to tackle this challenge utilizing nanotechnology to resolve the resistance mechanism caused by the tumor coverage.
